# Radiolabelling and preclinical characterisation of [^89^Zr]Zr-Df-ATG-101 bispecific to PD-L1/4–1BB

**DOI:** 10.1007/s00259-024-06742-6

**Published:** 2024-05-11

**Authors:** Zhipeng Cao, Christian Werner Wichmann, Ingrid Julienne Georgette Burvenich, Laura Danielle Osellame, Nancy Guo, Angela Rigopoulos, Graeme Joseph O’Keefe, Fiona Elizabeth Scott, Nirmal Lorensuhewa, Kevin Patrick Lynch, Andrew Mark Scott

**Affiliations:** 1 Tumour Targeting Laboratory, Olivia Newton-John Cancer Research Institute, Melbourne, Australia; 2 School of Cancer Medicine, La Trobe University, Melbourne, Australia; 3 Department of Molecular Imaging and Therapy, Austin Health, Melbourne, Australia; 4 School of Chemistry – Bio21 Institute, University of Melbourne, Melbourne, Australia; 5 Department of Medicine, University of Melbourne, Melbourne, Australia; 6 Antengene Biologics Limited, Shanghai, China

**Keywords:** PD-L1, 4-1BB, ATG-101, PET, Zirconium-89

## Abstract

**Purpose:**

ATG-101, a bispecific antibody that simultaneously targets the immune checkpoint PD-L1 and the costimulatory receptor 4-1BB, activates exhausted T cells upon PD-L1 crosslinking. Previous studies demonstrated promising anti-tumour efficacy of ATG-101 in preclinical models. Here, we labelled ATG-101 with ^89^Zr to confirm its tumour targeting effect and tissue biodistribution in a preclinical model. We also evaluated the use of immuno-PET to study tumour uptake of ATG-101 in vivo.

**Methods:**

ATG-101, anti-PD-L1, and an isotype control were conjugated with *p*-SCN-Deferoxamine (Df). The Df-conjugated antibodies were radiolabelled with ^89^Zr, and their radiochemical purity, immunoreactivity, and serum stability were assessed. We conducted PET/MRI and biodistribution studies on [^89^Zr]Zr-Df-ATG-101 in BALB/c nude mice bearing PD-L1-expressing MDA-MB-231 breast cancer xenografts for up to 10 days after intravenous administration of [^89^Zr]Zr-labelled antibodies. The specificity of [^89^Zr]Zr-Df-ATG-101 was evaluated through a competition study with unlabelled ATG-101 and anti-PD-L1 antibodies.

**Results:**

The Df-conjugation and [^89^Zr]Zr -radiolabelling did not affect the target binding of ATG-101. Biodistribution and imaging studies demonstrated biological similarity of [^89^Zr]Zr-Df-ATG-101 and [^89^Zr]Zr-Df-anti-PD-L1. Tumour uptake of [^89^Zr]Zr-Df-ATG-101 was clearly visualised using small-animal PET imaging up to 7 days post-injection. Competition studies confirmed the specificity of PD-L1 targeting in vivo.

**Conclusion:**

[^89^Zr]Zr-Df-ATG-101 in vivo distribution is dependent on PD-L1 expression in the MDA-MB-231 xenograft model. Immuno-PET with [^89^Zr]Zr-Df-ATG-101 provides real-time information about ATG-101 distribution and tumour uptake in vivo. Our data support the use of [^89^Zr]Zr-Df-ATG-101 to assess tumour and tissue uptake of ATG-101.

**Supplementary Information:**

The online version contains supplementary material available at 10.1007/s00259-024-06742-6.

## Introduction

Several inhibitory immune checkpoint proteins have been identified, along with their corresponding ligands found on various cells, including dendritic cells and tumour cells. These immune checkpoint proteins engage in interactions with their ligands, sending inhibitory signals to T cells, thus allowing tumours to escape immune surveillance [1]. Among these interactions, immunosuppressive effect induced by the interaction between programmed cell death protein 1 (PD-1) and programmed death-ligand 1 (PD-L1) is well-characterised in clinical immunotherapy. Multiple immune checkpoint inhibitors (ICIs) disrupting the PD-1/PD-L1 interaction have been successfully developed, exhibiting durable therapeutic benefits in many cancers, while only a subset of cancer patients benefit from ICIs monotherapy [2, 3].

In response to this limitation, combination therapies involving different ICIs are under active development, with the goal of enhancing the therapeutic efficacy of single agents. Additional benefits have been demonstrated when ICIs are combined with chemotherapy, radiotherapy, and small molecule targeted therapy [4]. Although ICIs often yield durable responses in cancer patients, adaptive resistance can develop over time. Blocking one immune checkpoint can induce the upregulation of alternative immune checkpoints on T cells [5–7]. Consequently, combination of two ICIs targeting different immune checkpoints exhibited synergistic effects, leading to improvements in progression-free survival and overall survival [8, 9]. Unfortunately, combination therapies involving two ICIs have typically been associated with a significantly higher incidence of adverse effects compared to monotherapy [10, 11]. To address these challenges and enhance both efficacy and safety, bispecific antibodies that simultaneously target two distinct antigens have been developed [12]. The affinity and valency of bispecific antibody arms can be tailored to minimise damage to normal cells.

4-1BB, also known as CD137 and TNFRS9, is an inducible costimulatory receptor expressed by activated T cells, monocytes, and natural killer (NK) cells [13]. Stimulation of 4-1BB on T cells activates various signalling pathways, resulting in increased cytokine secretion, enhanced T cell proliferation, improved T cell survival, and enhanced effector function [14, 15]. Agonistic antibodies targeting 4-1BB demonstrated promising anti-tumour activity in preclinical studies [16, 17]. While moderate anti-tumour responses have been observed in patients receiving 4-1BB agonistic antibodies, dose-limiting on-target-off-tumour hepatotoxicity was observed [18, 19]. To address this challenge, bispecific antibodies targeting 4-1BB and other anti-tumour targets have been developed, with the potential to minimize systemic toxicity of 4-1BB by limiting the costimulatory effect to tumour geography. For instance, a bispecific antibody targeting B7-H3/4-1BB can elicit a 4-1BB-dependent anti-tumour response in tumours expressing B7-H3, without causing systemic toxicity [20]. Another bispecific antibody targeting HER2/4-1BB demonstrated strong 4-1BB activation and anti-tumour effects in h4-1BB knock-in mice bearing HER2-positive tumours [21]. Combining 4-1BB agonism with ICI targeting PD-L1 resulted in increased CD8^+^ T cell infiltration and induced tumour regression in preclinical models [22, 23]. The bispecific antibody ATG-101, an anti-PD-L1 IgG1 molecule linked with two anti-4-1BB scFV molecules, has been developed and demonstrated potent anti-tumour efficacy in various preclinical models [24]. Importantly, this bispecific antibody exhibited good tolerance in non-human primates without significant toxicity.

The identification of target expression to select patients who are likely to respond to corresponding targeted therapies or immunotherapies is important in drug development and management of cancer patients. The use of radiolabelled molecules in conjunction with advanced in vivo bioimaging techniques such as positron emission tomography (PET) has been employed to examine in vivo expression of specific immune targets and has demonstrated great potential for patient selection [25–28]. In this study, we conjugated and radiolabelled ATG-101 bispecific antibody, the parental anti- PD-L1 antibody, and an isotype control antibody, with the positron-emitting isotope ^89^Zr. We demonstrated the suitability of [^89^Zr]Zr-Df-ATG-101 for in vivo bioimaging of PD-L1/4-1BB, supporting its potential application in clinical settings.

## Materials and methods

### Cell culture

MDA-MB-231 cells (ATCC, USA) were cultured in RPMI 1640 medium supplemented with 10% foetal calf serum (FCS). The human PD-L1-transfected HEK293 stable cell line (HEK293/PD-L1, GenScript #M00544) was cultured in Dulbecco modified Eagle medium (DMEM) supplemented with 10% FCS and 400 µg/mL G418. HEK293/4-1BB cell line (BPS Bioscience #60,691), expressing full-length human 4-1BB, was cultured in MEM medium supplemented with 10% FCS and 100 µg/mL Hygromycin B. All cell cultures were maintained at 37 °C with 5% CO_2_.

### Flow cytometry (FACS)

The binding of antibodies to PD-L1 and 4-1BB was assessed by flow cytometry using HEK293/PD-L1, HEK293/4-1BB, and MDA-MB-231 cell lines. Briefly, 1 × 10^6^ HEK293/PD-L1, HEK293/4-1BB, or MDA-MB-231 cell were incubated with 20 µg/mL of primary antibodies for 40 min at room temperature, followed by incubation with a phycoerythrin (PE)-conjugated goat anti-human IgG secondary antibody for 1 h at 4 °C. Flow analysis was performed using a FACS Canto II flow cytometer.

### Antibody conjugation and [^89^Zr]Zr-labelling

The bispecific antibody ATG-101 and parental anti-PD-L1 and anti-4-1BB were obtained from Antengene. All antibodies were conjugated with the bifunctional metal ion chelator, p-SCN-Deferoxamine (Df; Macrocyclics Inc.), at a 3 to 10-fold molar excess in sodium bicarbonate buffer (0.1 M, pH 9.0). The reaction mixture was incubated at room temperature for 1 h, followed by PD-10 gel filtration using 20 mM sodium succinate (pH 6.0), 8% sucrose, and 0.02% Tween 80. A solution of [^89^Zr]Zr-oxalate (0.05 M, Austin Health Cyclotron) was neutralized with sodium carbonate (0.1 M, pH 10.8) and diluted with reaction buffer (20 mM sodium succinate, pH 6, 0.02% Tween 80). Df-conjugated antibodies in formulation buffer were added to the neutralized ^89^Zr solution, and reaction mixture was incubated at room temperature for 15 min. Following incubation, purification was performed by PD-10 gel filtration with 20 mM sodium succinate (pH 6.0), 8% sucrose, and 0.02% Tween 80. Aliquots were stored at -80 °C until required.

### Determination of Df-to-mAb ratios for Df-conjugated antibodies

At the end of Df-mAb conjugation reaction, 25 µL of reaction mixture was added to 25 µL of 0.05 M succinic acid. Subsequently, 3 MBq neutralized [^89^Zr]Zr-oxalate was added to the mixture to label both free Df and Df-mAb in solution. Complete labelling was determined by instant thin layer chromatography (iTLC) using a citrate mobile phase (20 mM sodium citrate, pH 5.0). Labelled free Df and Df-mAb were determined and quantified by iTLC using a methanol/TFA mobile phase (4% trifluoracetic acid in 50% methanol). The Df-to-mAb ratios were then calculated by using the following formula: (reacting Df: mAb ratio) × ([^89^Zr]Zr-Df-mAb%)

### Immunoreactivity and scatchard analyses

The immunoreactive fraction of [^89^Zr]Zr-Df-ATG-101 for PD-L1 and 4-1BB was determined by linear extrapolation to binding at infinite antigen excess using a Lindmo assay [29]. [^89^Zr]Zr-Df-ATG-101 (20 ng) was added to 0–10 × 10^6^ HEK293/PD-L1 cells or 0–60 × 10^6^ HEK293/4-1BB cells in 1.0 ml of medium. The cells were incubated for 45 min at room temperature and pellets were measured in a gamma counter. Percentage binding was graphed against cell concentration, and immunoreactivities were calculated as the y-intercept of the inverse plot of both values. Scatchard analysis was used to calculate the association constant and the number of antibody molecules bound per cell.

### Ex vivo serum stability

Ex vivo serum stability was assessed by incubating 5.0 µg of [^89^Zr]Zr-Df-antibody in healthy donor human serum (100 µL) at 37 °C for a 7-day period. Radiochemical purity was determined by iTLC and SE-HPLC, and single-point immunoreactivity assays were performed at 0, 2, and 7 days of incubation.

### Animal model

In vivo characterisation was performed using the MDA-MB-231 xenograft murine model, given high PD-L1 expression on MDA-MB-231 cells [30]. Briefly, 10 × 10^6^ MDA-MB-23 cells were subcutaneously injected into the right flank of 5–6-week-old female BALB/c nude mice (Animal Research Centre, Australia), and tumour volume (TV) was monitored daily. TV was calculated by the following formula: (length × width^2^)/2. All animal studies were approved by the Austin Health Animal Ethics Committee and conducted in compliance with the Australian Code of Practice for the care and use of animals for scientific purposes.

### Biodistribution studies

BALB/c nude mice with established MDA-MB-231 xenografts (mean TV = 237.38 ± 44.83 mm^3^) received [^89^Zr]Zr-Df-ATG-101, [^89^Zr]Zr-Df-anti-PD-L1, or [^89^Zr]Zr-Df-huIgG1 isotype control (36 µg, 185 kBq) via intravenous injection. Groups of mice (*n* = 5 for each group) receiving [^89^Zr]Zr-Df-ATG-101 were sacrificed by over-inhalation of isoflurane anaesthesia and biodistribution was assessed on day 0 (2 h), 1, 2, 3, 5, 7, 10 post injection (p.i). Tissues, tumour, and blood were obtained for radioactive counting via a gamma counter and weighing. Mice receiving [^89^Zr]Zr-Df-anti-PD-L1 or [^89^Zr]Zr-Df-huIgG1 isotype control were assessed on day 2 and 7 p.i.

### In vivo competition and imaging studies

Groups of BALB/c nude mice (*n* = 5 for each group) with established MDA-MB-231 xenografts (mean TV = 230.38 ± 35.60 mm^3^) received intravenous injection of [^89^Zr]Zr-Df-huIgG1 isotype control (36 µg, 3.9 MBq), [^89^Zr]Zr-Df-anti-PD-L1 (36 µg, 3.9 MBq), [^89^Zr]Zr-Df-ATG-101 (36 µg, 3.9 MBq) alone or in combination with 1 mg of cold ATG-101, 1 mg of anti-PD-L1, or 1 mg of anti-4-1BB. Mice were imaged with positron emission tomography (PET) and magnetic resonance (MR) on day 2 and 7 using a small animal nanoPET/MR camera (nanoScan, Mediso). After imaging at each time point, five animals per group were analysed using a biodistribution study as described earlier. The co-registration of multi-modality scans and image analysis were performed using PMOD 3.8 (PMOD Technologies LLC).

### Immunohistochemistry (IHC) and haematoxylin and eosin staining (H&E)

Formalin fixed, paraffin embedded tumours were sectioned, then dewaxed in two washes of xylene, followed by rehydration in decreasing concentrations of ethanol. Antigen retrieval was performed in EDTA buffer pH 8.0 at 90 °C for 20 min. Slides were quenched with 3% hydrogen peroxide for 5 min, washed with water and blocked with 5% bovine serum albumin and 5% normal goat serum in Tris Buffered Saline with Tween 20 (TBST) for 1 h at room temperature. Slides were incubated with anti-PD-L1 (1:100; Cell Signalling, #13,684) in 5% normal goat serum in TBST overnight at 4 °C. Isotype control (rabbit IgG; Dako) was incubated alongside the primary antibody. Slides were incubated with anti-rabbit HRP secondary antibodies (Aligent) for 1 h at room temperature. Sections were thoroughly washed prior to counterstaining with Heamatoxylin followed by blueing in Scotts Tap water and dehydration in increasing ethanol percentages and two final washes in xylene. For haemotaxylin and eosin staining, samples were dewaxed and rehydrated, stained with haematoxylin, rinsed in water prior to blueing in Scotts Tap Water, rinsed again in water and stained in 0.1% alcoholic eosin. Images were acquired on an Aperio AT2 Slide Scanner (Leica Biosystems). Images were processed using Aperio ImageScope software (Leica Biosystems).

### Statistical analyses

All data analysis was performed using GraphPad Prism V8. For multiple comparisons, one-way ANOVA was used with Tukey’s multiple comparison test. Data are presented as mean ± SD, unless stated differently. Flowjo V10 was used for flow cytometry analysis.

## Results

### Conjugation and radiolabelling of ATG-101 and controls

**Fig. 1:**
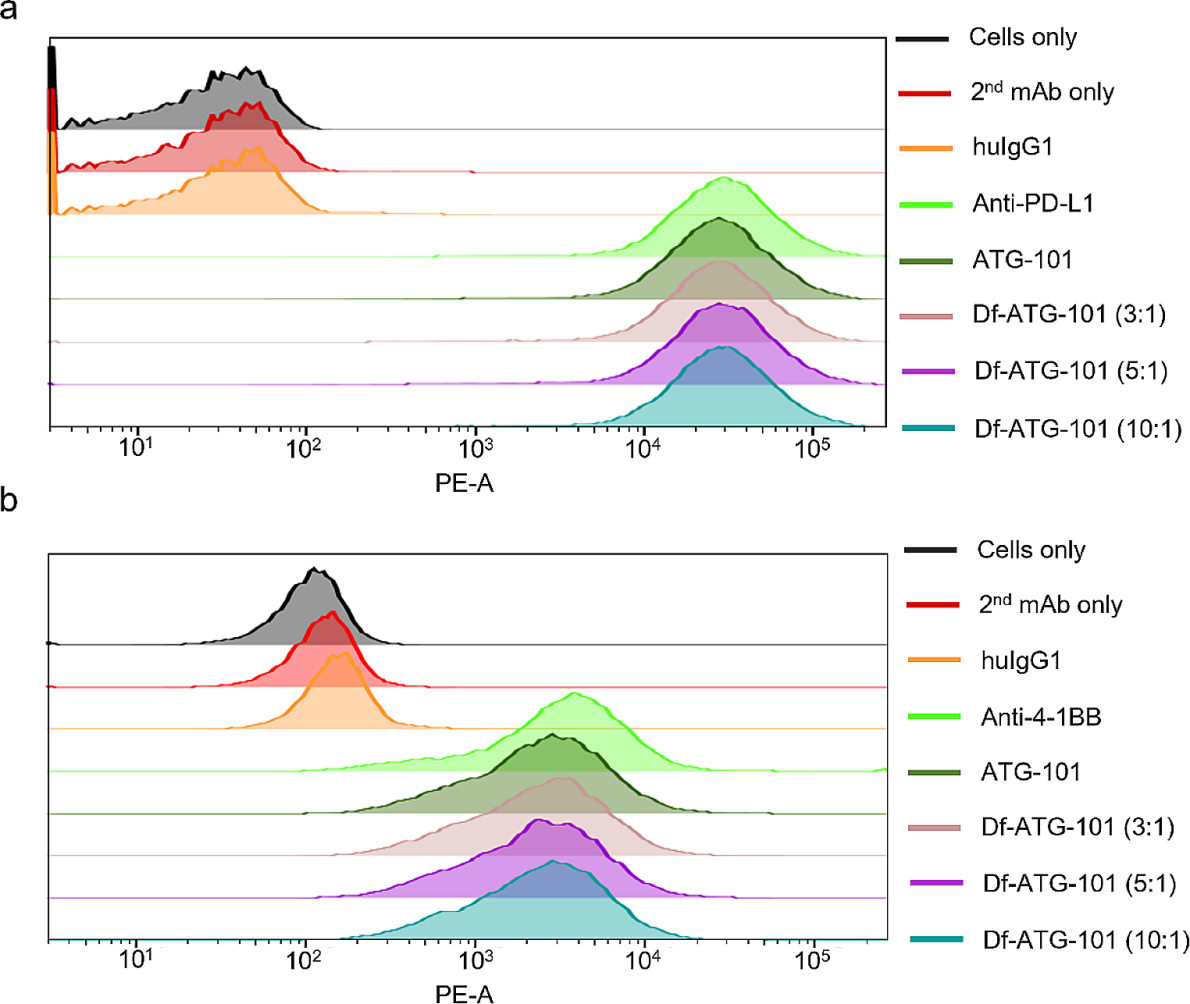
Flow cytometry of HEK293/PD-L1 and HEK293/4-1BB cells with ATG-101 and control antibodies. **a** HEK293/PD-L1 cells were stained with indicated antibodies that were detected with a PE-conjugated secondary antibody. **b** HEK293/4-1BB cells were stained with indicated antibodies that were detected with a PE-conjugated secondary antibody

The bifunctional metal ion chelator *p*-SCN-Bn-Deferoxamine (Df) was conjugated to ATG-101 and two monoclonal antibody controls including anti-PD-L1 and anti-4-1BB mAbs, yielding Df-ATG-101, Df-anti-PD-L1, and Df-anti-4-1BB. Given that chelator-antibody ratio can influence antibody characteristics such as stability and binding, we explored optimal Df-to-antibody ratio for preparation of Df-ATG-101. The reactions were performed at 3-, 5-, and 10-fold molar excess of Df to ATG-101, resulting in production of Df-ATG-101 (3:1), Df-ATG-101 (5:1), and Df-ATG-101 (10:1), with the final conjugated Df-to-mAb ratio of 0.62, 1.16, and 2.33, respectively. When incubated with HEK293/PD-L1 cells, anti-PD-L1 mAb and ATG-101 displayed identical binding levels (Fig. 1a). Importantly, all variants of Df-ATG-101 and ATG-101 exhibited equivalent binding to HEK293/PD-L1 cells, indicating that the conjugation process did not change ATG-101’s binding capacity to PD-L1 (Fig. 1a). The binding of ATG-101 to 4-1BB was confirmed using HEK293/4-1BB cells. ATG-101 and anti-4-1BB exhibited comparable binding to HEK293/4-1BB cells (Fig. 1b). All Df-ATG-101 variants demonstrated consistent binding profiles to ATG-101 using HEK293/4-1BB cell, indicating that Df conjugation to ATG-101 did not alter its binding affinity to 4-1BB.

Df-ATG-101 immunoconjugates with different Df-mAb ratios were then subjected to radiolabelling with ^89^Zr, yielding [^89^Zr]Zr-Df-ATG-101. Radiochemical yields, purities, and apparent specific activities of all [^89^Zr]Zr-Df-ATG-101 constructs were found to be comparable (Supplementary Table 1). To assess serum stability, [^89^Zr]Zr-Df-ATG-101 were incubated with human serum for up to 7 days. [^89^Zr]Zr-Df-ATG-101 (5:1) and [^89^Zr]Zr-Df-ATG-101 (10:1) displayed the highest levels of radiochemical purity over time. Compared to [^89^Zr]Zr-Df-ATG-101 (10:1), [^89^Zr]Zr-Df-ATG-101 (5:1) exhibited greater retention of binding to 4-1BB on day 7. Consequently, [^89^Zr]Zr-Df-ATG-101 (5:1) was employed for all following studies. The same Df-antibody ratio was applied to prepare all control antibodies in this study.

**Fig. 2:**
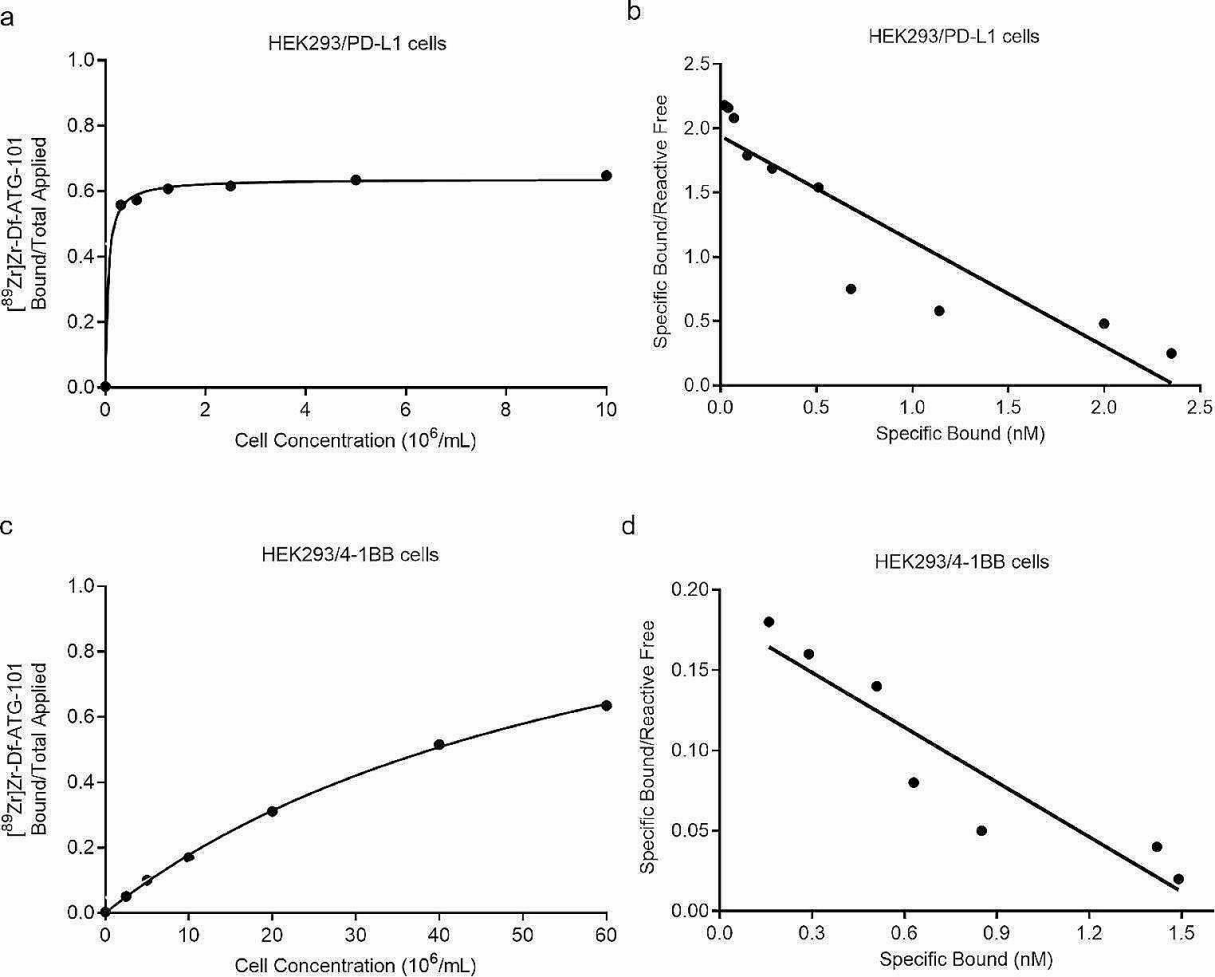
In vitro characterisation of PD-L1 and 4-1BB binding of [^89^Zr]Zr-Df-ATG-101. **a** Lindmo plots showing in vitro binding of [^89^Zr]Zr-Df-ATG-101 to increasing concentrations of HEK293/PD-L1 cells; **b** Scatchard plots of [^89^Zr]Zr-Df-ATG-101 binding to HEK293/PD-L1 cells; **c** Lindmo plots showing in vitro binding of [^89^Zr]Zr-Df-ATG-101 to HEK293/4-1BB cells; **d** Scatchard plots of [^89^Zr]Zr-Df-ATG-101 binding to HEK293/4-1BB cells

The binding characteristics of [^89^Zr]Zr-Df-ATG-101 to PD-L1 and 4-1BB were investigated using a Lindmo cell binding assay and Scatchard analysis (Fig. 2). Notably, [^89^Zr]Zr-Df-ATG-101 exhibited high affinity binding to both HEK293/PD-L1 (Fig. 2a and b) and HEK293/4-1BB cells (Fig. 2c and d), with binding affinities of 1.2 nM and 8.77 nM, respectively.

### Biodistribution of [^89^Zr]Zr-Df-ATG-101 and controls in mice bearing MDA-MB-231 tumours

**Fig. 3:**
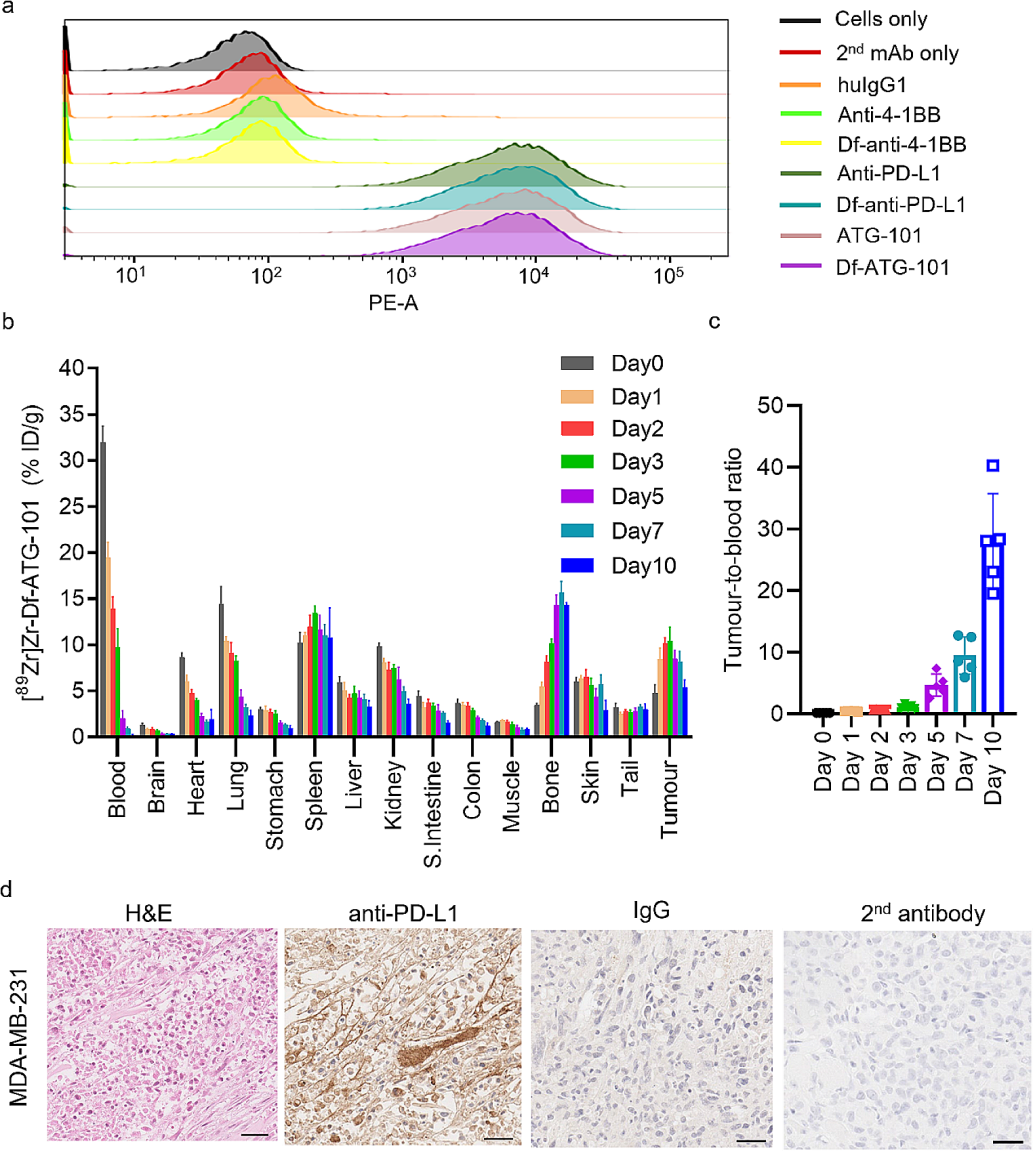
Biodistribution of [^89^Zr]Zr-Df-ATG-101 (185 kBq, 36 µg) in BALB/c nude mice bearing MDA-MB-231 tumours. **a** Flow cytometry showing PD-L1 binding of ATG-101 to MDA-MB-231 cells; **b** Biodistribution of [^89^Zr]Zr-Df-ATG-101 in BALB/c nude mice bearing MDA-MB-231 tumours; **c** Tumour-to-blood ratios of [^89^Zr]Zr-Df-ATG-101 over time. Bars indicate mean ± SD (*n* = 5). **d** Representative images for H&E and anti-PD-L1 IHC of MDA-MB-231 tumour, *n* = 3, scale bar = 50 μm. The rabbit IgG and 2nd antibody were used as controls

The expression status of PD-L1 varies among tumours from different patients, clinically classified as negative, low, or high PD-L1, based on the percentage of tumour cell staining: <1%, ≥ 1% and < 50%, or ≥ 50%, respectively [31]. The MDA-MB-231 cell line, originally derived from a female with metastatic breast cancer, is commonly used as a model to mimic human tumours with high PD-L1 expression, with a reported 97.5% of tumours cells being PD-L1 positive [32]. Given reported high level of PD-L1 expression in the MDA-MB-231 cells and tumours [30, 33, 34], this cell line was chosen for characterising [^89^Zr]Zr-Df-ATG-101 in vivo tumour targeting and biodistribution. Although ATG-101 cross-reacts with murine PD-L1, displaying a binding affinity of 1.58 nM, it does not cross-react with murine 4-1BB [24], and human 4-1BB knock-in mice were not available for investigation. Hence, biodistribution of [^89^Zr]Zr-Df-anti-4-1BB could not be investigated in this study. The binding of ATG-101 and anti-PD-L1 mAb to MDA-MB-231 cells was confirmed by FACS analysis (Fig. 3a). To explore the biodistribution of [^89^Zr]Zr-Df-ATG-101, mice bearing MDA-MB-231 tumours received intravenous administration of [^89^Zr]Zr-Df-ATG-101 (185 kBq, 36 µg) on day 0. The uptake of [^89^Zr]Zr-Df-ATG-101 in tumour peaked on day 3 (Fig. 3b). High uptake of [^89^Zr]Zr-Df-ATG-101 was also observed in normal organs, including spleen, lung, kidney, and bone. Similar to uptake observed in tumours, splenic uptake also peaked on day 3. High PD-L1 expression and uptake of anti-PD-L1 mAb in spleen was previously reported in both immunocompetent and immunocompromised murine models [35–37]. The uptake in lung and kidney followed a similar clearance pattern as blood (Fig. 3b), suggesting that uptake in these organs was non-specific. High bone uptake of [^89^Zr]Zr-Df-ATG-101 could be resulted from abundant PD-L1 expression in bone marrow cells. Given the murine PD-L1 cross-reactivity of the ATG-101, this model could provide a representative insight into the distribution of this antibody in humans. Additionally, [^89^Zr]Zr-labelled molecules could be metabolized, leading to release of free ^89^Zr with affinity for bones due to binding to hydroxyapatite [38]. Another study using a [^89^Zr]Zr-labelled anti-mouse PD-L1 also reported high bone uptake in tumour bearing mice [39]. In addition to targeting PD-L1, the deposition of [^89^Zr]Zr-Df-ATG-101 in spleen and bone marrow could be attributed to its interaction with FcRn in these tissues. The CH2 domain of Fc region of ATG-101 was mutated to abolish binding capacity to FcγRs while retaining the ability to bind to FcRn [24]. A FcRn distribution analysis by immunohistochemistry revealed high FcRn expression in normal tissue including spleen and bone marrow in mice [40]. The tumour-to-blood ratio of radioactivity increased over time (Fig. 3c), suggesting specificity of the tumour localisation. The PD-L1 positivity in MDA-MB-231 tumours was confirmed by IHC staining (Fig. 3d).

**Fig. 4:**
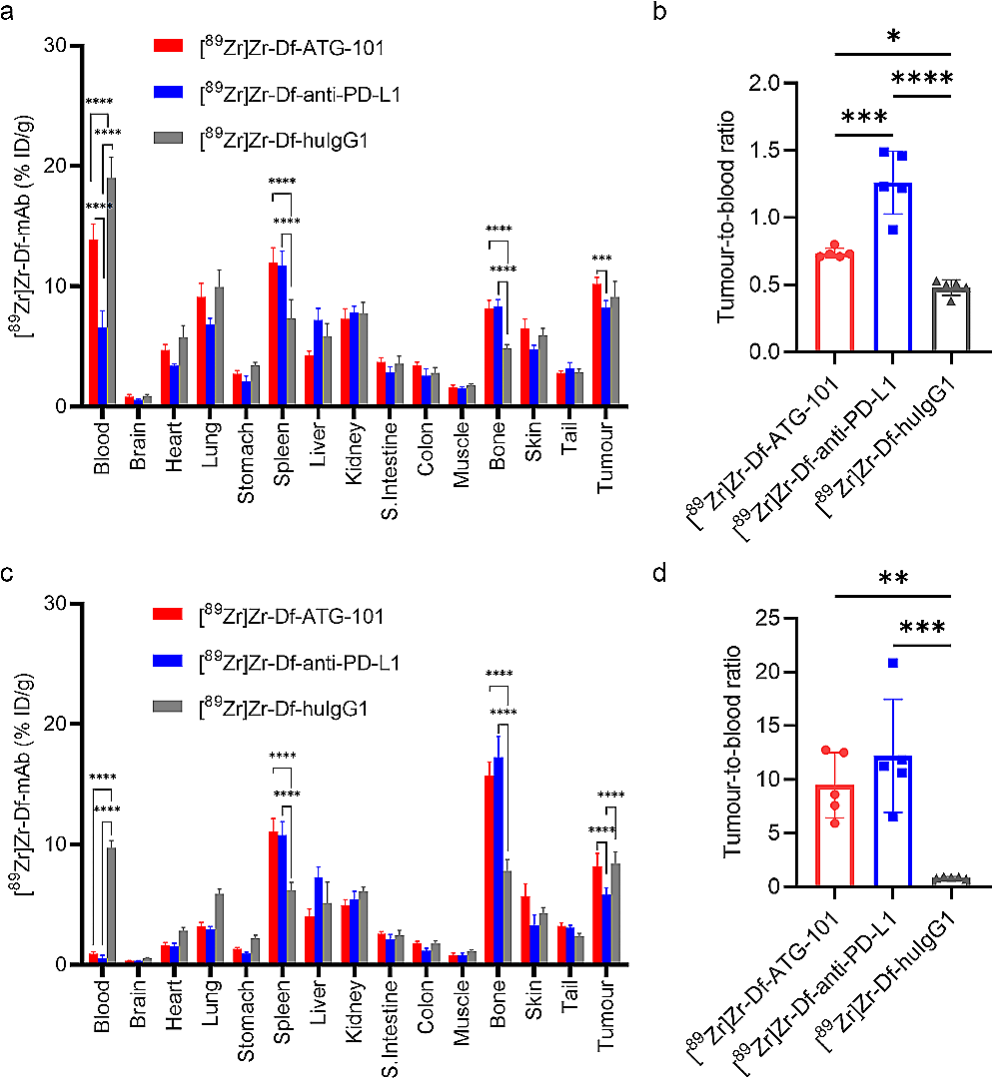
Biodistribution of [^89^Zr]Zr-Df-ATG-101, [^89^Zr]Zr-Df-anti-PD-L1, and [^89^Zr]Zr-Df-huIgG1 isotype control (36 µg, 3.9 MBq). **a** Biodistribution of indicated radiotracers on day 2 p.i; **b** Tumour-to-blood ratios on day 2 p.i; **c** Biodistribution of indicated radiotracers on day 7 p.i; **d** Tumour-to-blood ratios on day 7 p.i. Data are mean ± SD, *n* = 5 for each group. Comparations were made only for biodistributions in blood, spleen, bone, and tumours, *p < 0.05, **p < 0.01, ***p < 0.001, ****p < 0.0001

To further characterise the specificity of in vivo PD-L1 binding, biodistribution of [^89^Zr]Zr-Df-ATG-101 was compared to that of [^89^Zr]Zr-labelled isotype IgG control and [^89^Zr]Zr-Df-anti-PD-L1 on day 2 (Fig. 4a and b) and day 7 (Fig. 4c and d). [^89^Zr]Zr-Df-anti-PD-L1 exhibited a similar biodistribution profile to [^89^Zr]Zr-Df-ATG-101 (Fig. 4a and c), suggesting that distribution of [^89^Zr]Zr-Df-ATG-101 was mediated by PD-L1 binding in this model. In contrast to the PD-L1 targeting antibodies, blood retention of [^89^Zr]Zr-Df-huIgG1 control antibody was much higher. PD-L1 rich organs, such as spleen and bone, acted as an antigen sink for [^89^Zr]Zr-Df-ATG-101 and [^89^Zr]Zr-Df-anti-PD-L1. The absolute tumour uptake of [^89^Zr]Zr-Df-ATG-101 and [^89^Zr]Zr-Df-anti-PD-L1 was comparable to that of [^89^Zr]Zr-Df-huIgG1. However, this might be attributed to the biodistribution of [^89^Zr]Zr-Df-huIgG1 due to the absence of normal tissue targets for huIgG1, and high circulating blood levels (Fig. 4a and c). In contrast, the radioactivity of [^89^Zr]Zr-Df-ATG-101 and [^89^Zr]Zr-Df-anti-PD-L1 in the blood was relatively low, especially on day 7, while the tumour uptakes remained high. The tumour-to-blood ratio proves to be a superior parameter for assessing the uptake rate of radiotracers compared to tumour uptake alone [41, 42], as the background for PET imaging is largely derived from blood radioactivity. Specific tumour targeting effect of [^89^Zr]Zr-Df-ATG-101 and [^89^Zr]Zr-Df-anti-PD-L1 is evident from the tumour-to-blood ratios (Fig. 4b and d).

### Competition studies of [^89^Zr]Zr-Df-ATG-101 in mice bearing MDA-MB-231 tumours

**Fig. 5:**
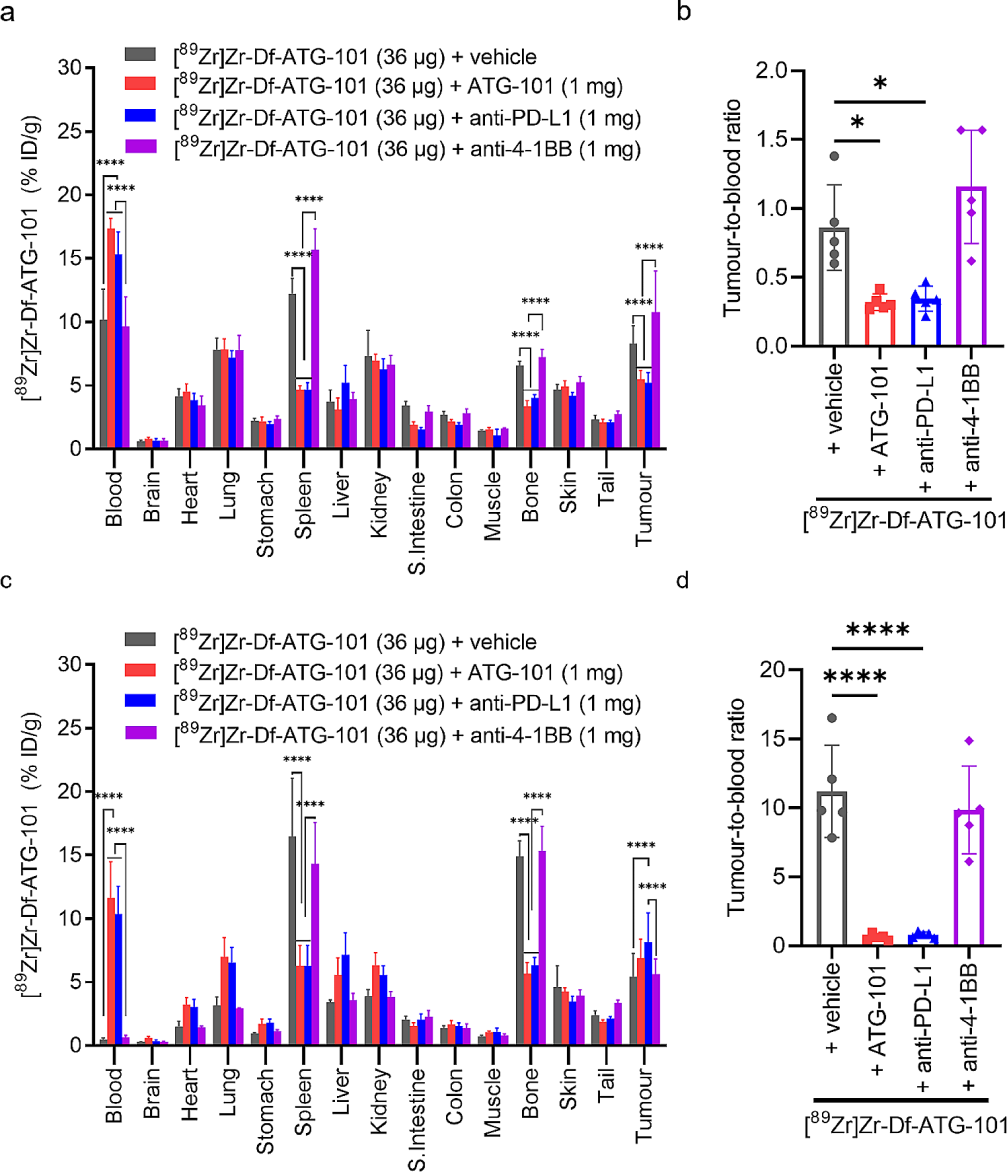
In vivo competition study of [^89^Zr]Zr-Df-ATG-101 using cold antibodies. **a** Biodistribution of [^89^Zr]Zr-Df-ATG-101 with or without competing antibodies on day 2 p.i; **b** Tumour-to-blood ratios on day 2 p.i; **c** Biodistribution of [^89^Zr]Zr-Df-ATG-101 with or without competing antibodies on day 7 p.i; **d** Tumour-to-blood ratios on day 7 p.i. Bars indicate mean ± SD, *n* = 5. Comparations were made for biodistributions in blood, spleen, bone, and tumours, *p < 0.05, ****p < 0.0001

A competition study was conducted to confirm the specificity of [^89^Zr]Zr-Df-ATG-101 biodistribution in vivo. Mice bearing MDA-MB-231 tumours were administered [^89^Zr]Zr-Df-ATG-101 (36 µg, 3.9 MBq) alone or in addition of 1 mg of non-radiolabelled antibodies including ATG-101, anti-4-1BB, or anti-PD-L1 (Fig. 5). Uptake in spleen and bone was significantly decreased with addition of excess unlabelled ATG-101 and anti-PD-L1 but not with anti-4-1BB mAb (Fig. 5a and c). This confirms that biodistribution of [^89^Zr]Zr-Df-ATG-101 is primarily mediated by the PD-L1 binding feature of ATG-101 antibody in this model. Although ATG-101 is an anti-PD-L1/4-1BB bispecific antibody, it exclusively binds to human 4-1BB and does not cross-react with murine 4-1BB. As anticipated, the presence of excess anti-4-1BB mAb did not significantly alter the biodistribution of [^89^Zr]Zr-Df-ATG-101 due to the lack of antigen in the mouse. Significantly lower tumour-to-blood ratios were observed with excess unlabelled ATG-101 or anti-PD-L1 antibodies (Fig. 5b and d), further confirming specific binding of [^89^Zr]Zr-Df-ATG-101 to PD-L1. Due to the sink effect, high expression of PD-L1 in normal organs like spleen can result in the entrapment of the antibody within these organs when a low dose of the radiotracer was administrated. Consequently, this could lead to a low level of radioactivity in blood. However, when an excess of cold competing antibody was co-administered, these antigen sinks became saturated, allowing more unbound radioactivity to remain in the blood. Interestingly, although competing ATG-101 and anti-PD-L1 reduced tumour uptake of [^89^Zr]Zr-Df-ATG-101 on day 2, the blocking effect was not evident on day 7 (Fig. 5b). The binding and blockade of PD-L1 by competing antibodies in normal organs like spleen might progressively increase over time. This could potentially make MDA-MB-231 tumour cells more accessible to circulating [^89^Zr]Zr-Df-ATG-101 after passage through the spleen. Another possibility is that the ATG-101 bispecific antibody may have target-mediated drug disposition effect, resulting in nonlinear clearance. A similar observation was reported in another study investigating anti-PD-L1 biodistribution in a murine model of melanoma [43]. Despite these factors, the tumour-to-blood ratios suggest that the blocking effect remained significant on day 7.

### PET/MRI imaging studies with [^89^Zr]Zr-Df-ATG-101 in mice bearing MDA-MB-231 tumours

**Fig. 6:**
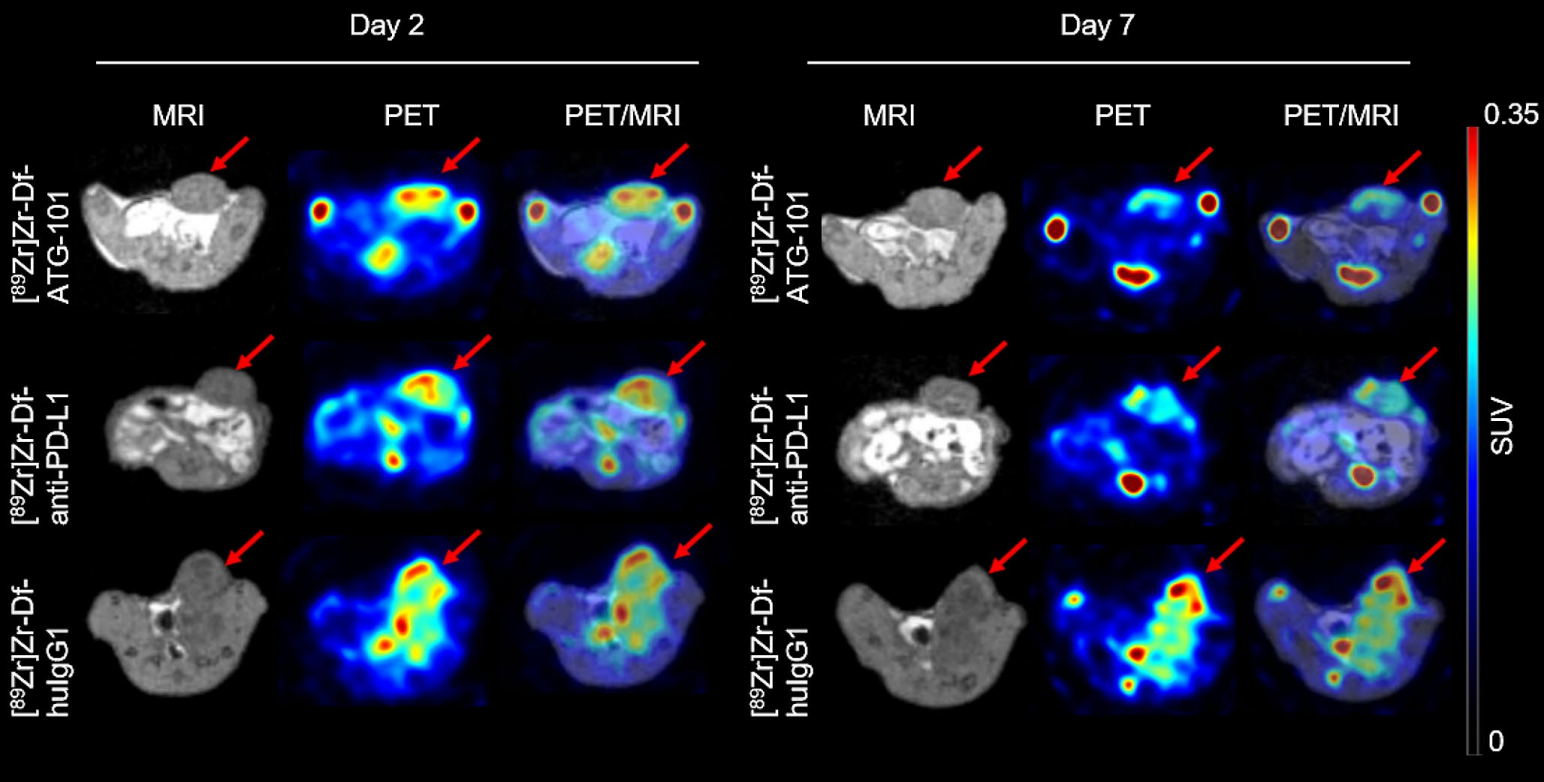
PET/MRI transaxial images of BALB/c nude mice bearing MDA-MB-231 tumours injected with [^89^Zr]Zr-labelled antibodies (36 µg, 3.9 MBq). MRI, PET, and PET/MRI overlay images were captured on day 2 and day 7 p.i of indicated radiotracers. Red arrows indicate the location of tumours. SUV, standard uptake value

Biodistribution of [^89^Zr]Zr-Df-ATG-101 in the MDA-MB-231 model was visualised using PET/MRI. Each mouse bearing MDA-MB-231 tumours received [^89^Zr]Zr-labelled antibodies (36 µg, 3.9 MBq) followed by PET/MRI scans on day 2 and day 7 p.i. The PET and PET/MRI images demonstrated uptake in tumour and bone tissue on day 2 and day 7 p.i (Fig. 6). [^89^Zr]Zr-Df-anti-PD-L1 radiotracer displayed similar imaging profiles to [^89^Zr]Zr-Df-ATG-101. Bone uptake of [^89^Zr]Zr-Df-ATG-101 and [^89^Zr]Zr-Df-anti-PD-L1 increased on day 7, while the radioactivity in blood was lower compared with day 2 (Fig. 6). Due to the absence of a binding target for [^89^Zr]Zr-Df-huIgG1, its level remained high in bloodstream on both day 2 and day 7, as demonstrated by the biodistribution study. The high uptake values of [^89^Zr]Zr-Df-huIgG1 in tumours shown by PET could be attributed to high blood flow in tumour tissues, which are known for their angiogenic characteristics. These PET results align with the findings from the biodistribution analysis (Fig. 4). Post-mortem biodistribution analysis after PET imaging on day 7 showed good alignment with the image analysis (Figure S1).

## Discussion

Immunotherapy has significantly advanced the treatment of cancer patients, with T cells playing a crucial role in efficacy of immunotherapies like ICIs and adoptive cell therapies. The state of T cells within tumour microenvironment is closely linked to effectiveness of these therapies. T cell exhaustion, characterised by gradual loss of effector function and self-renewal capacity, is inversely correlated with the success of immunotherapy [44]. Activation of 4-1BB signalling in exhausted T cells has been shown to promote their proliferation and differentiation [45, 46], making 4-1BB an attractive target for developing immune based therapeutics, especially in tumours that display a relatively immunosuppressed tumour microenvironment. However, anti-4-1BB antibodies have exhibited dose-limiting on-target-off-tumour hepatotoxicity in the clinic [47], which could be mediated through liver macrophages activated by nonspecific hepatic memory CD8^+^ T cells triggered by anti-4-1BB [48]. Different strategies have been proposed to bypass the dose-limiting toxicities. A common strategy is to develop bispecific antibodies that bind both 4-1BB and another target specific to tumours, confining most of the 4-1BB costimulatory activity to tumour geography. These bispecific antibodies, such as 4-1BB/HER2 [49], 4-1BB/B7-H3 [20], and 4-1BB/PD-L1 [24], have shown potential in preclinical and clinical settings. Not only can the bispecific antibody enrich 4-1-BB agonism in tumours and reduce its level in liver, but it may also improve the efficacy of monotherapies through an additive or synergistic effect.

The ATG-101 bispecific antibody, designed to target both PD-L1 and 4-1BB, activates 4-1BB positive T cells in a PD-L1-crosslinking dependent manner. It exhibited potent anti-tumour effect in various preclinical models, including those sensitive and resistant to anti-PD-L1 treatments [24]. Cynomolgus monkeys receiving ATG-101 did not show significant hepatoxicity based on levels of alanine transaminase and aspartate transaminase in serum [24]. Phase I clinical trials (NCT04986865, NCT05490043) for ATG-101 are currently ongoing. This study aimed to develop radiolabelled ATG-101 for immuno-PET imaging, which can be used to select patients for ATG-101 therapy in clinical trials. At a time when identification of optimal dosing for further studies is under close regulatory scrutiny [50], exploration of the biodistribution of [^89^Zr]Zr-Df-ATG-101 across a range of potentially active doses could provide additional information to select a dose that optimizes PD-L1 antagonism and 4-1BB agonism, whilst minimizing the potential for systemic toxicity. We have shown that radiolabelled [^89^Zr]Zr-Df-ATG-101 are highly suited for clinical translation. This radiotracer maintained its integrity, high affinity and immunoreactivity in vitro for cell lines expressing PD-L1 or 4-1BB. Additionally, it exhibited excellent serum stability and in vivo targeting properties.

In preclinical PD-L1 xenograft biodistribution studies, the expected uptake of [^89^Zr]Zr-Df-ATG-101 and [^89^Zr]Zr-Df-anti-PD-L1 in spleen and bone marrow aligns with reported PD-L1 expression in these organs. This pattern is likely to be consistent in humans, supported by the findings from the [^89^Zr]-atezolizumab (anti-PD-L1) first-in-human study [28]. Given significantly high tumour-to-blood ratio of [^89^Zr]Zr-Df-ATG-101 and [^89^Zr]Zr-Df-anti-PD-L1, especially on day 7, we believe that these agents have potential to diagnose PD-L1 expression in tumours and provide satisfactory imaging properties in patients. The murine model was established in BALB/c nude mice lacking thymus and T cells, hence, thymus uptake of [^89^Zr]Zr-Df-ATG-101 was not investigated in this study. As 4-1BB is mainly expressed in T cells and ATG-101 does not bind murine 4-1BB, the biodistribution and in vivo characteristics of [^89^Zr]Zr-Df-ATG-101 were driven by its PD-L1 binding, supported by the similar distribution profile of [^89^Zr]Zr-Df-anti-PD-L1 in this model, and the results of competition with excess unlabelled anti-PD-L1. In the future, it will be worthwhile to investigate [^89^Zr]Zr-Df-ATG-101 biodistribution in a human 4-1BB knock-in preclinical model. However, the considerably greater affinity of ATG-101 for PD-L1 over 4-1BB suggests that the data seen in these experiments will remain relevant to clinical trials.

The PET/MRI studies provided results consistent with the biodistribution and competition studies. Immuno-PET imaging allowed for clear visualization of uptake in tumours and normal organs, underscoring the potential of [^89^Zr]Zr-Df-ATG-101 for patient selection and optimal dose determination in clinical settings. When competition with excessive unlabelled ATG-101 or anti-PD-L1 antibodies was introduced, reduced uptake in normal tissues and organs was observed, confirming the PD-L1 specificity of ATG-101.

Tumour PD-L1 expression is the most widely used biomarker for selecting patients to receive anti-PD1/PD-L1 treatments [51]. For example, atezolizumab has been approved for treating locally recurrent unresectable or metastatic triple-negative breast cancer with more than 1% PD-L1 staining cells in total tumour area [52]. Currently, clinical evaluation of PD-L1 expression primarily relies on immunohistochemical assays. However, the use of these immunohistochemical assays in clinic presents several inherent challenges including the requirements of different companion diagnostic assays, high inter-assay heterogeneity on performance and cut-off points, and the potential difference in PD-L1 expression between different tumours in the same patient. These disadvantages may contribute to the lack of correlation between PD-L1 expression and treatment response in clinic. In contrast, the immuno-PET-based PD-L1 diagnostic method can be standardised by establishing a reproducible threshold of uptake to predict outcomes. Furthermore, it offers additional advantages, such as being a non-invasive approach that can visualise systemic PD-L1 expression. This could be particularly valuable in patients with inoperable or late-stage cancers suitable for anti-PD-L1 therapy.

Several radiolabelled antibodies against PD-L1 have been developed for detecting PD-L1 expression in tumours and assess response to PD-L1 blockade therapy [28, 53–55]. For instance, [^89^Zr]Zr-atezolizumab immuno-PET demonstrated high uptake in PD-L1 positive tumours and spleens of cancer patients [28]. Clinical responses of these patients were better correlated with the immuno-PET signal than with immunohistochemistry-based predictive biomarkers [28]. In a preclinical study, [^89^Zr]Zr-avelumab exhibited tumour uptake in PD-L1 positive tumours and high splenic uptake [56, 57]. The biodistribution of a bispecific antibody is determined by its binding to both targets. As ATG-101 concurrently binds to PD-L1 and 4-1BB, the distribution of [^89^Zr]Zr-anti-PD-L1 antibodies may not accurately reflect the actual distribution of ATG-101 in patients. Therefore, the development of [^89^Zr]Zr-ATG-101 is required to guide patient selection of clinical trials for this antibody. To the best of our knowledge, this [^89^Zr]Zr-Df-ATG-101 antibody is the first radiolabelled bispecific antibody targeting PD-L1 and 4-1BB. Although this study was limited by the absence of human 4-1BB expression in the murine model, our Lindmo-Scatchard analysis demonstrated that [^89^Zr]Zr-Df-ATG-101 binds to 4-1BB with high affinity, validating its target binding characteristics.

## Conclusion

In conclusion, this preclinical study demonstrates the feasibility of imaging biodistribution and tumour uptake of the ATG-101 bispecific antibody using the ^8^[^89^Zr]Zr-Df-ATG-101 immuno-PET. These findings provide compelling evidence for potential application of [^89^Zr]Zr-Df-ATG-101 in bioimaging clinical trials for cancer patients and are likely to be of relevance in the early development of other bispecific antibodies targeting immune checkpoints. This novel approach holds promise for advancing patient selection, dose determination and patient monitoring in the context of immunotherapy trials.

### Electronic supplementary material

Below is the link to the electronic supplementary material.

Supplementary Material 1

## Data Availability

Data to support the findings in this study are included in the manuscript or its supplementary information. Additional data can be made available from the corresponding author upon reasonable request.

## References

[1] Wykes MN, Lewin SR. Immune checkpoint blockade in infectious diseases. Nat Rev Immunol. 2018;18:91–104. 10.1038/nri.2017.112. 28990586 10.1038/nri.2017.112 PMC5991909

[2] Powles T, Plimack ER, Soulières D, Waddell T, Stus V, Gafanov R, et al. Pembrolizumab plus Axitinib versus sunitinib monotherapy as first-line treatment of advanced renal cell carcinoma (KEYNOTE-426): extended follow-up from a randomised, open-label, phase 3 trial. Lancet Oncol. 2020;21:1563–73. 10.1016/s1470-2045(20)30436-8. 33284113 10.1016/s1470-2045(20)30436-8

[3] Adams S, Loi S, Toppmeyer D, Cescon DW, De Laurentiis M, Nanda R, et al. Pembrolizumab monotherapy for previously untreated, PD-L1-positive, metastatic triple-negative breast cancer: cohort B of the phase II KEYNOTE-086 study. Ann Oncol. 2019;30:405–11. 10.1093/annonc/mdy518. 30475947 10.1093/annonc/mdy518

[4] Vafaei S, Zekiy AO, Khanamir RA, Zaman BA, Ghayourvahdat A, Azimizonuzi H, et al. Combination therapy with immune checkpoint inhibitors (ICIs); a new frontier. Cancer Cell Int. 2022;22:2. 10.1186/s12935-021-02407-8. 34980128 10.1186/s12935-021-02407-8 PMC8725311

[5] Koyama S, Akbay EA, Li YY, Herter-Sprie GS, Buczkowski KA, Richards WG, et al. Adaptive resistance to therapeutic PD-1 blockade is associated with upregulation of alternative immune checkpoints. Nat Commun. 2016;7:10501. 10.1038/ncomms10501. 26883990 10.1038/ncomms10501 PMC4757784

[6] Curran MA, Montalvo W, Yagita H, Allison JP. PD-1 and CTLA-4 combination blockade expands infiltrating T cells and reduces regulatory T and myeloid cells within B16 melanoma tumors. Proc Natl Acad Sci U S A. 2010;107:4275–80. 10.1073/pnas.0915174107. 20160101 10.1073/pnas.0915174107 PMC2840093

[7] Gao J, Ward JF, Pettaway CA, Shi LZ, Subudhi SK, Vence LM, et al. VISTA is an inhibitory immune checkpoint that is increased after ipilimumab therapy in patients with prostate cancer. Nat Med. 2017;23:551–5. 10.1038/nm.4308. 28346412 10.1038/nm.4308 PMC5466900

[8] Larkin J, Chiarion-Sileni V, Gonzalez R, Grob JJ, Rutkowski P, Lao CD, et al. Five-year survival with combined Nivolumab and Ipilimumab in Advanced Melanoma. N Engl J Med. 2019;381:1535–46. 10.1056/NEJMoa1910836. 31562797 10.1056/NEJMoa1910836

[9] Larkin J, Chiarion-Sileni V, Gonzalez R, Grob JJ, Cowey CL, Lao CD, et al. Combined Nivolumab and Ipilimumab or Monotherapy in untreated melanoma. N Engl J Med. 2015;373:23–34. 10.1056/NEJMoa1504030. 26027431 10.1056/NEJMoa1504030 PMC5698905

[10] Boutros C, Tarhini A, Routier E, Lambotte O, Ladurie FL, Carbonnel F, et al. Safety profiles of anti-CTLA-4 and anti-PD-1 antibodies alone and in combination. Nat Reviews Clin Oncol. 2016;13:473–86. 10.1038/nrclinonc.2016.58. 10.1038/nrclinonc.2016.58 27141885

[11] Wolchok JD, Chiarion-Sileni V, Gonzalez R, Rutkowski P, Grob J-J, Cowey CL, et al. Overall survival with combined Nivolumab and Ipilimumab in Advanced Melanoma. N Engl J Med. 2017;377:1345–56. 10.1056/NEJMoa1709684. 28889792 10.1056/NEJMoa1709684 PMC5706778

[12] Dahlén E, Veitonmäki N, Norlén P. Bispecific antibodies in cancer immunotherapy. Therapeutic Adv Vaccines Immunotherapy. 2018;6:3–17. 10.1177/2515135518763280. 10.1177/2515135518763280 PMC5933537 29998217

[13] Kim KM, Kim HW, Kim JO, Baek KM, Kim JG, Kang CY. Induction of 4-1BB (CD137) expression by DNA damaging agents in human T lymphocytes. Immunology. 2002;107:472–9. 10.1046/j.1365-2567.2002.01538.x. 12460192 10.1046/j.1365-2567.2002.01538.x PMC1782822

[14] Sanchez-Paulete AR, Labiano S, Rodriguez-Ruiz ME, Azpilikueta A, Etxeberria I, Bolaños E, et al. Deciphering CD137 (4-1BB) signaling in T-cell costimulation for translation into successful cancer immunotherapy. Eur J Immunol. 2016;46:513–22. 10.1002/eji.201445388. 26773716 10.1002/eji.201445388

[15] Sabbagh L, Pulle G, Liu Y, Tsitsikov EN, Watts TH. ERK-Dependent Bim Modulation downstream of the 4-1BB-TRAF1 Signaling Axis is a critical mediator of CD8 T cell survival in Vivo1. J Immunol. 2008;180:8093–101. 10.4049/jimmunol.180.12.8093. 18523273 10.4049/jimmunol.180.12.8093

[16] Melero I, Shuford WW, Newby SA, Aruffo A, Ledbetter JA, Hellström KE, et al. Monoclonal antibodies against the 4-1BB T-cell activation molecule eradicate established tumors. Nat Med. 1997;3:682–5. 10.1038/nm0697-682. 9176498 10.1038/nm0697-682

[17] Gauttier V, Judor JP, Le Guen V, Cany J, Ferry N, Conchon S. Agonistic anti-CD137 antibody treatment leads to antitumor response in mice with liver cancer. Int J Cancer. 2014;135:2857–67. 10.1002/ijc.28943. 24789574 10.1002/ijc.28943

[18] Segal NH, He AR, Doi T, Levy R, Bhatia S, Pishvaian MJ, et al. Phase I study of single-Agent Utomilumab (PF-05082566), a 4-1BB/CD137 agonist, in patients with Advanced Cancer. Clin Cancer Res. 2018;24:1816–23. 10.1158/1078-0432.Ccr-17-1922. 29549159 10.1158/1078-0432.Ccr-17-1922

[19] Segal NH, Logan TF, Hodi FS, McDermott D, Melero I, Hamid O, et al. Results from an Integrated Safety Analysis of Urelumab, an agonist Anti-CD137 monoclonal antibody. Clin Cancer Res. 2017;23:1929–36. 10.1158/1078-0432.Ccr-16-1272. 27756788 10.1158/1078-0432.Ccr-16-1272

[20] You G, Lee Y, Kang YW, Park HW, Park K, Kim H, et al. B7-H3×4-1BB bispecific antibody augments antitumor immunity by enhancing terminally differentiated CD8(+) tumor-infiltrating lymphocytes. Sci Adv. 2021;7. 10.1126/sciadv.aax3160. 10.1126/sciadv.aax3160 PMC7810375 33523913

[21] Song MY, Lee E-J, Chung H, Lee Y, Park YB, Jee MH, et al. Abstract 6524: a novel HER2/4-1BB bispecific antibody, YH32367 (ABL105) shows potent anti-tumor effect through tumor-directed T cell activation. Cancer Res. 2020;80:6524. 10.1158/1538-7445.Am2020-6524. 10.1158/1538-7445.Am2020-6524

[22] Qu QX, Zhu XY, Du WW, Wang HB, Shen Y, Zhu YB, et al. 4-1BB Agonism Combined with PD-L1 Blockade increases the number of tissue-resident CD8 + T cells and facilitates Tumor Abrogation. Front Immunol. 2020;11:577. 10.3389/fimmu.2020.00577. 32391001 10.3389/fimmu.2020.00577 PMC7193033

[23] Wang Y, Zhang X, Xu C, Nan Y, Fan J, Zeng X, et al. Targeting 4-1BB and PD-L1 induces potent and durable antitumor immunity in B-cell lymphoma. Front Immunol. 2022;13:1004475. 10.3389/fimmu.2022.1004475. 36544785 10.3389/fimmu.2022.1004475 PMC9762552

[24] Yuwen H, Li T, Ren Y, Hoenemann D, Mei J, Shan B, et al. 893 ATG-101, a novel PD-L1/4–1BB bispecific antibody, augments anti-tumor immunity through immune checkpoint inhibition and PDL1-directed 4–1BB activation. J Immunother Cancer. 2021;9:A936–7. 10.1136/jitc-2021-SITC2021.893. 10.1136/jitc-2021-SITC2021.893

[25] Burvenich IJG, Goh YW, Guo N, Gan HK, Rigopoulos A, Cao D, et al. Radiolabelling and preclinical characterization of (89)Zr-Df-radiolabelled bispecific anti-PD-L1/TGF-βRII fusion protein bintrafusp alfa. Eur J Nucl Med Mol Imaging. 2021;48:3075–88. 10.1007/s00259-021-05251-0. 33608805 10.1007/s00259-021-05251-0

[26] Wichmann CW, Poniger S, Guo N, Roselt P, Rudd SE, Donnelly PS, et al. Automated radiosynthesis of [89Zr]Zr-DFOSq-Durvalumab for imaging of PD-L1 expressing tumours in vivo. Nucl Med Biol. 2023;120–121:108351. 10.1016/j.nucmedbio.2023.108351. 37224789 10.1016/j.nucmedbio.2023.108351

[27] Parakh S, Lee ST, Gan HK, Scott AM. Radiolabeled antibodies for Cancer Imaging and Therapy. Cancers. 2022;14:1454. 35326605 10.3390/cancers14061454 PMC8946248

[28] Bensch F, van der Veen EL, Lub-de Hooge MN, Jorritsma-Smit A, Boellaard R, Kok IC, et al. ^89^Zr-atezolizumab imaging as a non-invasive approach to assess clinical response to PD-L1 blockade in cancer. Nat Med. 2018;24:1852–8. 10.1038/s41591-018-0255-8. 30478423 10.1038/s41591-018-0255-8

[29] Lindmo T, Boven E, Cuttitta F, Fedorko J, Bunn PA Jr. Determination of the immunoreactive fraction of radiolabeled monoclonal antibodies by linear extrapolation to binding at infinite antigen excess. J Immunol Methods. 1984;72:77–89. 10.1016/0022-1759(84)90435-6. 6086763 10.1016/0022-1759(84)90435-6

[30] Zheng Y, Fang YC, Li J. PD-L1 expression levels on tumor cells affect their immunosuppressive activity. Oncol Lett. 2019;18:5399–407. 10.3892/ol.2019.10903. 31612048 10.3892/ol.2019.10903 PMC6781757

[31] Lin G, Fan X, Zhu W, Huang C, Zhuang W, Xu H, et al. Prognostic significance of PD-L1 expression and tumor infiltrating lymphocyte in surgically resectable non-small cell lung cancer. Oncotarget. 2017;8:83986–94. 10.18632/oncotarget.20233. 29137398 10.18632/oncotarget.20233 PMC5663570

[32] Darga EP, Dolce EM, Fang F, Kidwell KM, Gersch CL, Kregel S, et al. PD-L1 expression on circulating tumor cells and platelets in patients with metastatic breast cancer. PLoS ONE. 2021;16:e0260124. 10.1371/journal.pone.0260124. 34780566 10.1371/journal.pone.0260124 PMC8592410

[33] Rom-Jurek E-M, Kirchhammer N, Ugocsai P, Ortmann O, Wege AK, Brockhoff G. Regulation of programmed death Ligand 1 (PD-L1) expression in breast Cancer cell lines in Vitro and in Immunodeficient and Humanized Tumor mice. Int J Mol Sci. 2018;19:563. 29438316 10.3390/ijms19020563 PMC5855785

[34] Zhang R, Yang Y, Dong W, Lin M, He J, Zhang X et al. D-mannose facilitates immunotherapy and radiotherapy of triple-negative breast cancer via degradation of PD-L1. Proceedings of the National Academy of Sciences. 2022;119:e2114851119. doi:10.1073/pnas.2114851119. 10.1073/pnas.2114851119 PMC8872783 35181605

[35] Josefsson A, Nedrow JR, Park S, Banerjee SR, Rittenbach A, Jammes F, et al. Imaging, Biodistribution, and Dosimetry of Radionuclide-labeled PD-L1 antibody in an Immunocompetent mouse model of breast Cancer. Cancer Res. 2016;76:472–9. 10.1158/0008-5472.Can-15-2141. 26554829 10.1158/0008-5472.Can-15-2141 PMC4715915

[36] Chatterjee S, Lesniak WG, Gabrielson M, Lisok A, Wharram B, Sysa-Shah P, et al. A humanized antibody for imaging immune checkpoint ligand PD-L1 expression in tumors. Oncotarget. 2016;7:10215–27. 10.18632/oncotarget.7143. 26848870 10.18632/oncotarget.7143 PMC4891115

[37] Giesen D, Broer LN, Lub-de Hooge MN, Popova I, Howng B, Nguyen M, et al. Probody Therapeutic Design of 89Zr-CX-072 promotes Accumulation in PD-L1–Expressing tumors compared to normal murine lymphoid tissue. Clin Cancer Res. 2020;26:3999–4009. 10.1158/1078-0432.Ccr-19-3137. 31953313 10.1158/1078-0432.Ccr-19-3137

[38] Abou DS, Ku T, Smith-Jones PM. In vivo biodistribution and accumulation of 89Zr in mice. Nucl Med Biol. 2011;38:675–81. 10.1016/j.nucmedbio.2010.12.011. 21718943 10.1016/j.nucmedbio.2010.12.011 PMC4527328

[39] Krache A, Fontan C, Pestourie C, Bardiès M, Bouvet Y, Payoux P, et al. Preclinical pharmacokinetics and Dosimetry of an 89Zr labelled Anti-PDL1 in an Orthotopic Lung Cancer Murine Model. Front Med. 2022;8. 10.3389/fmed.2021.741855. 10.3389/fmed.2021.741855 PMC8841431 35174180

[40] Latvala S, Jacobsen B, Otteneder MB, Herrmann A, Kronenberg S. Distribution of FcRn Across Species and tissues. J Histochem Cytochem. 2017;65:321–33. 10.1369/0022155417705095. 28402755 10.1369/0022155417705095 PMC5625855

[41] van den Hoff J, Oehme L, Schramm G, Maus J, Lougovski A, Petr J, et al. The PET-derived tumor-to-blood standard uptake ratio (SUR) is superior to tumor SUV as a surrogate parameter of the metabolic rate of FDG. EJNMMI Res. 2013;3:77. 10.1186/2191-219x-3-77. 24267032 10.1186/2191-219x-3-77 PMC4175513

[42] Hofheinz F, Hoff Jvd, Steffen IG, Lougovski A, Ego K, Amthauer H, et al. Comparative evaluation of SUV, tumor-to-blood standard uptake ratio (SUR), and dual time point measurements for assessment of the metabolic uptake rate in FDG PET. EJNMMI Res. 2016;6:53. 10.1186/s13550-016-0208-5. 27334609 10.1186/s13550-016-0208-5 PMC4917506

[43] Nedrow JR, Josefsson A, Park S, Ranka S, Roy S, Sgouros G. Imaging of programmed cell death Ligand 1: impact of protein concentration on distribution of Anti-PD-L1 SPECT agents in an Immunocompetent Murine Model of Melanoma. J Nucl Med. 2017;58:1560–6. 10.2967/jnumed.117.193268. 28522738 10.2967/jnumed.117.193268 PMC5632734

[44] Chi X, Luo S, Ye P, Hwang W-L, Cha J-H, Yan X, et al. T-cell exhaustion and stemness in antitumor immunity: characteristics, mechanisms, and implications. Front Immunol. 2023;14. 10.3389/fimmu.2023.1104771. 10.3389/fimmu.2023.1104771 PMC9986432 36891319

[45] Pichler AC, Carrié N, Cuisinier M, Ghazali S, Voisin A, Axisa PP, et al. TCR-independent CD137 (4-1BB) signaling promotes CD8(+)-exhausted T cell proliferation and terminal differentiation. Immunity. 2023;56:1631–e4810. 10.1016/j.immuni.2023.06.007. 37392737 10.1016/j.immuni.2023.06.007 PMC10649891

[46] Long AH, Haso WM, Shern JF, Wanhainen KM, Murgai M, Ingaramo M, et al. 4-1BB costimulation ameliorates T cell exhaustion induced by tonic signaling of chimeric antigen receptors. Nat Med. 2015;21:581–90. 10.1038/nm.3838. 25939063 10.1038/nm.3838 PMC4458184

[47] Chester C, Sanmamed MF, Wang J, Melero I. Immunotherapy targeting 4-1BB: mechanistic rationale, clinical results, and future strategies. Blood. 2018;131:49–57. 10.1182/blood-2017-06-741041. 29118009 10.1182/blood-2017-06-741041

[48] Wang J, Zhao W, Cheng L, Guo M, Li D, Li X, et al. CD137-Mediated pathogenesis from Chronic Hepatitis to Hepatocellular Carcinoma in Hepatitis B Virus-Transgenic mice. J Immunol. 2010;185:7654–62. 10.4049/jimmunol.1000927. 21059892 10.4049/jimmunol.1000927 PMC3601909

[49] Hinner MJ, Aiba RSB, Jaquin TJ, Berger S, Dürr MC, Schlosser C, et al. Tumor-localized Costimulatory T-Cell Engagement by the 4-1BB/HER2 bispecific antibody-Anticalin Fusion PRS-343. Clin Cancer Res. 2019;25:5878–89. 10.1158/1078-0432.Ccr-18-3654. 31138587 10.1158/1078-0432.Ccr-18-3654

[50] Murphy R, Halford S, Symeonides SN. Project Optimus, an FDA initiative: considerations for cancer drug development internationally, from an academic perspective. Front Oncol. 2023;13:1144056. 10.3389/fonc.2023.1144056. 36937434 10.3389/fonc.2023.1144056 PMC10020863

[51] Doroshow DB, Bhalla S, Beasley MB, Sholl LM, Kerr KM, Gnjatic S, et al. PD-L1 as a biomarker of response to immune-checkpoint inhibitors. Nat Reviews Clin Oncol. 2021;18:345–62. 10.1038/s41571-021-00473-5. 10.1038/s41571-021-00473-5 33580222

[52] Twomey JD, Zhang B. Cancer Immunotherapy Update: FDA-Approved checkpoint inhibitors and Companion Diagnostics. Aaps j. 2021;23:39. 10.1208/s12248-021-00574-0. 33677681 10.1208/s12248-021-00574-0 PMC7937597

[53] Lv G, Sun X, Qiu L, Sun Y, Li K, Liu Q, et al. PET imaging of Tumor PD-L1 expression with a highly specific nonblocking single-domain antibody. J Nucl Med. 2020;61:117–22. 10.2967/jnumed.119.226712. 31253743 10.2967/jnumed.119.226712 PMC6954462

[54] Brown EL, DeWeerd RA, Zidel A, Pereira PMR. Preclinical antibody-PET imaging of PD-L1. Front Nuclear Med. 2022;2. 10.3389/fnume.2022.953202. 10.3389/fnume.2022.953202 PMC11440863 39354977

[55] Bouleau A, Nozach H, Dubois S, Kereselidze D, Chevaleyre C, Wang C-I, et al. Optimizing Immuno-PET Imaging of Tumor PD-L1 expression: pharmacokinetic, Biodistribution, and dosimetric comparisons of ^89^Zr-Labeled Anti-PD-L1 antibody formats. J Nucl Med. 2022;63:1259–65. 10.2967/jnumed.121.262967. 34933891 10.2967/jnumed.121.262967 PMC9364342

[56] Jagoda EM, Vasalatiy O, Basuli F, Opina ACL, Williams MR, Wong K, et al. Immuno-PET imaging of the programmed cell Death-1 ligand (PD-L1) using a Zirconium-89 labeled therapeutic antibody, Avelumab. Mol Imaging. 2019;18:1536012119829986. 10.1177/1536012119829986. 31044647 10.1177/1536012119829986 PMC6498777

[57] Li M, Ehlerding EB, Jiang D, Barnhart TE, Chen W, Cao T, et al. In vivo characterization of PD-L1 expression in breast cancer by immuno-PET with (89)Zr-labeled avelumab. Am J Transl Res. 2020;12:1862–72. 32509182 PMC7270013

