# Supplementary material for: Radiolabelling and preclinical characterisation of [89Zr]Zr-Df-ATG-101 bispecific to PD-L1/4–1BB
Source: Eur J Nucl Med Mol Imaging. 2024 May 11;51(11):3202–14. doi: 10.1007/s00259-024-06742-6 (PMC11368977; doi:10.1007/s00259-024-06742-6)
Supplement: Supplementary file 1 — Supplementary Material 1 [file 259_2024_6742_MOESM1_ESM.docx]

**Radiolabelling and preclinical characterisation of [^89^Zr]Zr-Df-ATG-101 bispecific to PD-L1/4–1BB**

Zhipeng Cao^1,2,3*^, Christian Werner Wichmann^1,2,3,4*^, Ingrid Julienne Georgette Burvenich^1,2*^, Laura Danielle Osellame^1,2^, Nancy Guo^1^, Angela Rigopoulos^1^, Graeme Joseph O’Keefe^3,5^, Fiona Elizabeth Scott^1,2^, Nirmal Lorensuhewa^6^, Kevin Patrick Lynch^6^, Andrew Mark Scott^1,2,3,5^

^1^Tumour Targeting Laboratory, Olivia Newton-John Cancer Research Institute, Melbourne, Australia

^2^School of Cancer Medicine, La Trobe University, Melbourne, Australia

^3^Department of Molecular Imaging and Therapy, Austin Health, Melbourne, Australia

^4^School of Chemistry – Bio21 Institute, University of Melbourne, Melbourne, Australia

^5^Department of Medicine, University of Melbourne, Melbourne, Australia

^6^Antengene Biologics Limited

*Authors contributed equally

For correspondence or reprints, contact Andrew M. Scott ([Andrew.Scott@onjcri.org.au](mailto:Andrew.Scott@onjcri.org.au))

**Supplementary Table 1** Characteristics and Serum Stability of [^89^Zr]Zr-Df-ATG-101.

| *In vitro* assessment | | [^89^Zr]Zr-Df-ATG-101 (3:1) | [^89^Zr]Zr-Df-ATG-101 (5:1) | [^89^Zr]Zr-Df-ATG-101 (10:1) |
| --- | --- | --- | --- | --- |
| Df-to-mAb ratio | | 0.62 | 1.16 | 2.33 |
| Radiochemical yield (%) | | 79.6 | 79.1 | 77.5 |
| Apparent specific activity (MBq/nmol) | | 18.0 | 17.1 | 18.2 |
| Radiochemical purity (%) | Day 0 | 100 | 100 | 100 |
|  | Day 2 | 85.63 | 94.83 | 96.99 |
|  | Day 7 | 78.01 | 91.67 | 92.78 |
| PD-L1 binding (%) | Day 0 | 82.75 | 82.41 | 88.52 |
|  | Day 2 | 61.58 | 70.47 | 75.95 |
| 4-1BB binding (%) | Day 0 | 11.03 | 10.03 | 10.05 |
|  | Day 2 | 5.16 | 7.16 | 7.06 |
|  | Day 7 | 5.63 | 8.22 | 6.18 |


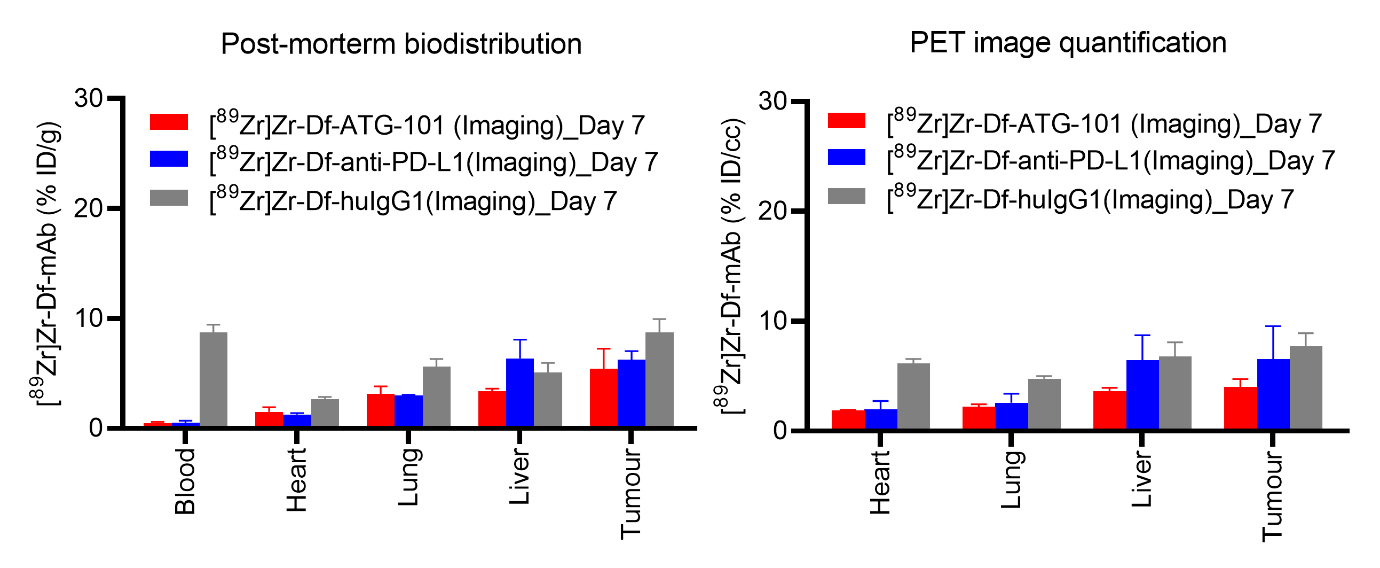


**Figure S1.** Post-mortem tissue biodistribution analysis (left) and PET imaging quantification (right) of mice from imaging study. The uptake in heart quantified by PET image analysis was a total uptake of heart and blood.
